# Preverbal trauma as a history-dependent response architecture: integrating bioelectrical, epigenetic, and developmental mechanisms

**DOI:** 10.3389/fnsys.2026.1862960

**Published:** 2026-10-26

**Authors:** Jarett Hancock, Deborah Becht-Buss

**Affiliations:** Oregon State University-Cascades, Bend, OR, United States

**Keywords:** biological embedding, developmental plasticity, early-life stress, epigenetic regulation, history-dependent responsivity, intrinsic excitability, preverbal trauma, stress neurobiology

## Abstract

Preverbal trauma occurs while neural, endocrine, regulatory, and stress-responsive systems are still developing, allowing early experience to influence later function even when autobiographical or linguistic access to the original event is limited. This article proposes a history-dependent response architecture as a mechanistic framework for understanding such developmental embedding. Rather than treating preverbal trauma as a memory stored in a distinct somatic substrate, a persistent abnormal resting membrane potential, or a single enduring biological state, the framework proposes that developmental experience can alter the evolving biological organization through which later inputs are transformed into physiological and behavioral responses. Evidence from early-life stress and developmental models is integrated across intrinsic neuronal physiology, synaptic organization, endocrine signaling, activity-dependent transcription and chromatin regulation, glial processes, and circuit maturation. These mechanisms can interact across development without requiring the process that initially produces a change to remain the mechanism that later expresses its consequences. Persistence may involve continued pathway involvement, structural or circuit remodeling, regulatory priming, or compensation, while in some cases the biological substrate carrying the developmental effect may itself change across development. This perspective predicts that systems with similar resting or behavioral outputs may nevertheless differ in latent biological configuration and therefore diverge when exposed to a standardized later challenge. Such differences may appear in response threshold, gain, recruitment, adaptation, coordination, or recovery and may vary with developmental timing, sex, genotype, exposure structure, cell population, and subsequent environment. The framework therefore shifts experimental emphasis from identifying a single persistent biological scar to measuring developmental trajectories and input-response functions across time and context. Longitudinal, challenge-based, and causal designs can distinguish this account from simpler cumulative-exposure or persistent-variable explanations, making the proposed architecture testable, refinable, and rejectable.

## 1 Introduction

### 1.1 Scope, terminology, and the translational problem

Human development begins long before experience can be organized into language. Infants learn associations, respond to caregivers, and adapt to patterns of safety and threat before they can later describe those experiences as part of an autobiographical narrative. Preverbal trauma therefore raises a developmental question rather than a simple question of memory. Early experiences can be encoded even when later spontaneous retrieval is limited, and infantile amnesia does not imply that the original experience was never learned or that it left no lasting biological consequence (Akers et al., 2014; Alberini and Travaglia, 2017; Guskjolen et al., 2018). The relevant distinction is between learning, later accessibility, and the developmental effects an experience may have on systems that are still maturing.

This paper uses preverbal trauma to describe consequential adversity occurring before mature linguistic and autobiographical organization is available. It does not assume that such experience bypasses memory and is instead stored in a separate somatic substrate, nor that an inaccessible episodic representation must remain intact. Instead, the focus is on biological embedding. Early experience occurs while stress-regulatory systems, synaptic organization, intrinsic neuronal properties, gene regulation, and large-scale circuits are still developing. An experience can therefore influence how these systems are constructed and how they later` respond, even when the original event cannot be narrated or naturally retrieved.

Much of the mechanistic evidence relevant to this problem comes from research on early-life stress, or ELS. Human preverbal trauma and experimental ELS are not equivalent constructs. Rodent maternal separation, limited bedding, altered maternal care, or other developmental manipulations do not reproduce the subjective experience of human trauma. Their value is that they allow developmental processes relevant to the human problem to be experimentally isolated, measured, and manipulated. Within this framework, preverbal trauma can be understood as a particularly consequential manifestation of the broader phenomenon of early-life biological embedding. It occupies a related developmental problem space to experimental ELS while referring to human experiences whose timing, intensity, duration, relational context, predictability, or regulatory consequences may produce enduring vulnerability. This framing allows animal ELS research to inform candidate mechanisms without treating experimental adversity as equivalent to human trauma or every early stressor as traumatic.

The central object of the theory is a history-dependent response architecture. This refers to the biological organization produced through development that influences how present input is transformed into present response. This organization can be expressed through history-dependent parameters distributed across intrinsic membrane physiology, synaptic organization, endocrine signaling, activity-dependent transcription, chromatin regulation, glial remodeling, and network maturation. The framework further allows the biological processes through which a developmental effect is expressed or maintained to change over time. Persistence of developmental influence therefore does not require one continuously abnormal variable or the indefinite persistence of the mechanism that initially produced the change. A system may appear relatively typical at rest yet respond differently when challenged because earlier experience altered the architecture through which that challenge is processed (McCrory and Viding, 2015; McCrory et al., 2017).

The theory developed here asks how early adversity changes those response rules and how such changes can remain biologically consequential after the initiating environment has ended. The proposed contribution is not the general claim that early adversity can have lasting effects, that preverbal experience can matter without later autobiographical recall, or that developing systems are calibrated by experience. Rather, the framework integrates experimentally supported electrical, synaptic, endocrine, epigenetic, glial, and network mechanisms into a mechanistically explicit account of history-dependent development. It asks where changes in neuronal physiology enter this process, how they interact with established endocrine and gene-regulatory mechanisms, how developmental influence may be expressed or maintained through different biological processes across development, and why some consequences may become most apparent when the system is challenged rather than under resting conditions. By specifying these relationships and the measurements needed to distinguish them from simpler accounts of persistent abnormality, the framework generates testable predictions about how developmental history alters later responsivity. Section 2 begins with the first part of that problem by examining why the effect of experience depends on the developmental state in which it occurs (Figure 1).

**Figure 1:**
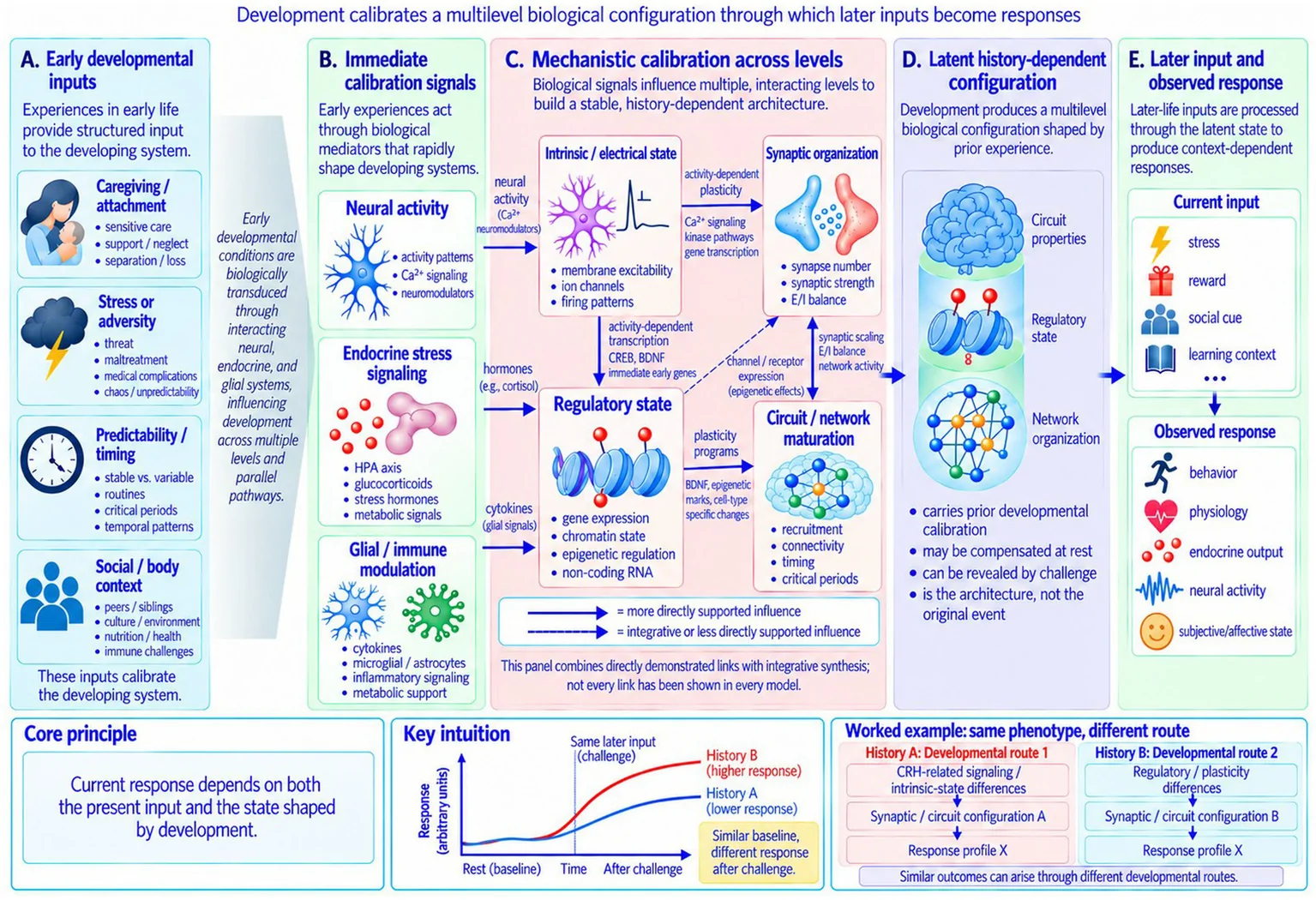
Building a latent response architecture from early experience.

## 2 Developmental calibration: why timing changes what experience does

### 2.1 Caregiver and environment as components of infant regulation

During early development, the caregiver is part of the infant’s regulatory environment. Contact, nursing, separation and reunion, maternal presence, and the predictability of caregiving conditions occur while neural and endocrine systems are still developing. Human developmental models similarly treat caregiving and broader environmental conditions as contributors to the development of stress physiology and self-regulation rather than as influences acting on an already completed nervous system (Blair and Raver, 2012; Rilling and Young, 2014; Lyons-Ruth and Jacobvitz, 2016). In rodents, variation in maternal care is associated with later differences in HPA responsivity and hippocampal glucocorticoid-receptor regulation (Weaver et al., 2004), while experimentally augmented maternal care alters excitatory input onto hypothalamic CRH neurons and subsequent regulation of CRH expression (Korosi et al., 2010; Singh-Taylor et al., 2018). Maternal presence can also suppress corticosterone responses and alter what infant rats learn from an otherwise similar sensory event (Moriceau and Sullivan, 2006). Caregiving is therefore not simply background context but patterned, multidimensional input contributing to the physiological conditions under which developing neural and stress-regulatory systems are repeatedly activated and continue to mature (Blair and Raver, 2012).

The effect of an experience also depends on when it occurs. Stress-sensitive systems do not mature synchronously. The amygdala, hippocampus, prefrontal cortex, HPA axis, and their interconnections follow different developmental trajectories, and consequences associated with adversity can vary with both age at exposure and age at measurement (Fenoglio et al., 2006; Gunnar and Quevedo, 2007; Lupien et al., 2009; Pechtel and Pizzagalli, 2011; Teicher and Samson, 2016; Tottenham and Sheridan, 2010). Developmental timing therefore changes the biological context in which an input is encountered because the circuits available for recruitment, endocrine conditions under which learning occurs, and forms of cellular and synaptic plasticity capable of responding are not constant across maturation (Figure 1).

Bitzenhofer et al. (2021) provide a causal example of why this matters. Transiently increasing coordinated activity in medial prefrontal layer 2/3 pyramidal neurons from P7–P11, during active cortical network formation, produced later alterations in excitation/inhibition organization, gamma synchronization, and prefrontal-dependent behavior. Importantly, altering activity during a later developmental window did not reproduce the same adult effects (Bitzenhofer et al., 2021). This experiment does not establish that early-life stress produces the same phenotype or operates through the same manipulation. Rather, it demonstrates the relevant developmental principle: transient alteration of neural activity during circuit formation can redirect aspects of subsequent maturation. Developmental timing should therefore be treated as a moderator of the mechanisms proposed in this framework rather than mapped onto a fixed sequence of mechanistic stages (Figure 2).

**Figure 2:**
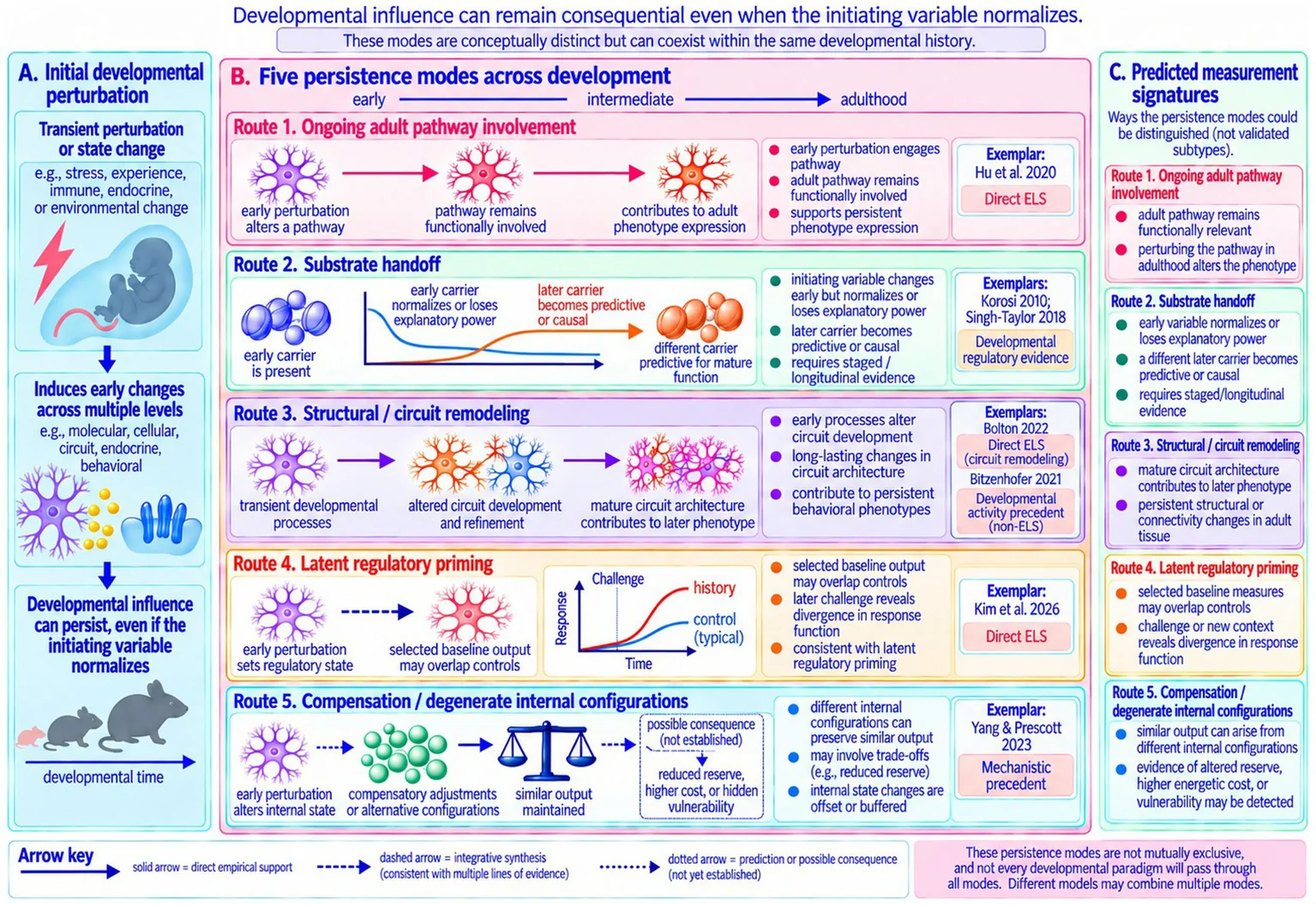
Persistence modes and substrate handoff.

### 2.2 Infant attachment learning as a comparative developmental proof of principle

Infant rat attachment learning provides an unusually clear demonstration that a similar conditioning relation can produce different learned outcomes depending on developmental and endocrine state. During the early attachment-sensitive period, odor learning is supported primarily by the olfactory bulb, locus coeruleus, and anterior piriform cortex rather than the adult-like amygdala-dependent fear-learning configuration. Under these conditions, pairing a novel odor with mild shock can produce learned preference rather than avoidance. As development proceeds and corticosterone becomes capable of recruiting amygdala participation, the same conditioning procedure can instead support aversion learning (Sullivan and Holman, 2010).

Moriceau and Sullivan (2006) demonstrated that this developmental transition remains strongly context dependent. In transitional-age pups, odor-shock conditioning in the mother’s absence activated the amygdala and produced odor avoidance, whereas conditioning in the mother’s presence produced preference without comparable amygdala activation. Maternal presence reduced pup corticosterone, and experimentally restoring corticosterone, including within the amygdala, reinstated odor aversion despite maternal presence (Moriceau and Sullivan, 2006). Thus, sensory input and conditioning contingency alone did not determine the learned response. Developmental stage, endocrine state, and caregiver context influenced which circuitry participated and which behavioral output followed.

Early caregiving conditions may also alter when particular circuits become functionally engaged. Sullivan and Holman (2010) review an insufficient-bedding caregiving paradigm in which altered maternal behavior and elevated pup corticosterone were associated with amygdala-dependent odor aversion at an age when normally reared pups continued to show age-typical preference learning. Because this inference is drawn here from review-level evidence rather than an independently evaluated primary experiment, it should be interpreted as evidence that stressful caregiving can alter developmental gating rather than as establishing a general rule that adversity accelerates fear-system maturation (Sullivan and Holman, 2010).

The translational value of this model is deliberately narrow. Rat olfaction is not an analogue of human language, and the olfactory bulb is not a substitute for the human prefrontal cortex. The shared principle is developmental: consequential learning occurs through the neural, endocrine, and regulatory systems available at the time. Human preverbal experience likewise occurs before later language-supported autobiographical organization and mature regulatory circuitry are fully available, without implying an absence of early learning or memory, as established in Section 1. The comparative inference is therefore not one of sensory or anatomical homology but of developmental dependence: experience is processed within the biological architecture available when it occurs.

### 2.3 Calibration, adaptation, and nonmonotonic outcomes

Developmental change following adversity should not be described exclusively as damage. Developmental and adaptive-calibration models describe emerging stress regulation as responsive to environmental conditions and allow multiple developmental trajectories rather than a single continuum from normal to progressively greater hyperreactivity (Blair and Raver, 2012; Del Giudice et al., 2011). Adaptation in this sense is descriptive rather than a judgment that the resulting phenotype is healthy, desirable, or cost free. A configuration shaped under one set of developmental conditions may therefore have different functional consequences when later conditions change (Blair and Raver, 2012; Del Giudice et al., 2011).

Direct experimental evidence also argues against assuming that greater early stress necessarily produces monotonically greater later hyperreactivity. Qin et al. (2019) exposed mice to one hour of maternal separation per day from PND3–21 and later subjected males to chronic social defeat stress. Earlier maternal separation attenuated later stress-associated anxiety-like behavior and reduced the associated increases in glutamatergic transmission and intrinsic excitability of basolateral amygdala (BLA) principal neurons, while maternal separation alone produced little change in several measured adult outcomes (Qin et al., 2019). This provides evidence for a stress-inoculation-like effect within one defined paradigm. It does not demonstrate that maternal separation is generally beneficial, that repeated adversity produces resilience, or that any particular spacing of exposure was responsible for the later phenotype (Figure 3).

**Figure 3:**
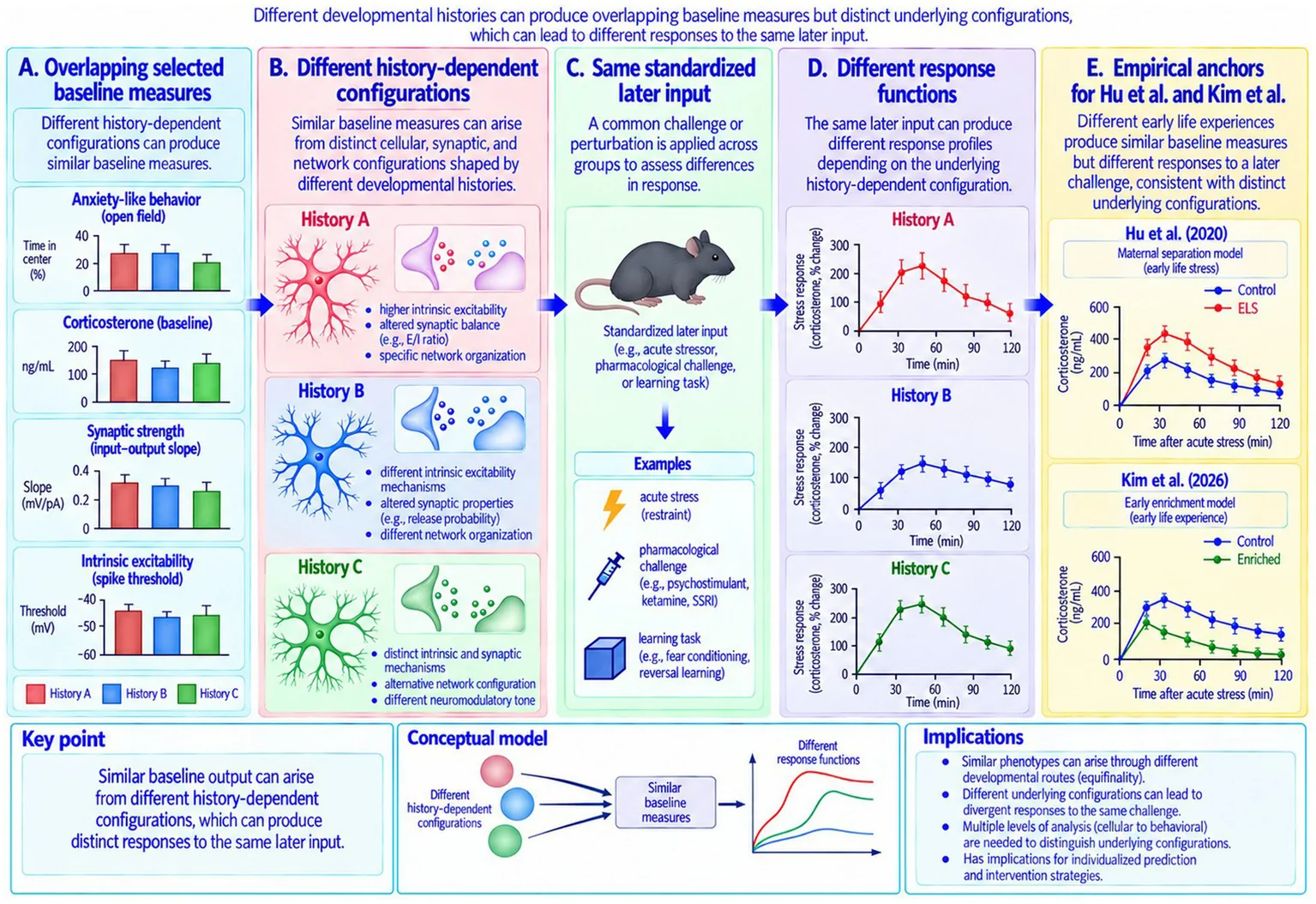
Similar baseline output can arise from distinct history-dependent states. The response curves are schematic illustrations of hypothetical developmental configurations and their predicted responses to later challenges, rather than quantitative reproductions of experimental findings. The early-enrichment model represents a theoretical extrapolation inspired by mechanisms described in Hu et al. (2020) and Kim et al. (2026), neither of which directly tested the illustrated enrichment paradigm. These studies serve as empirical anchors for the broader principle that developmental history can alter subsequent physiological responsivity.

These findings argue against representing developmental history solely as event count or cumulative “dose.” Intensity, duration, timing, repetition, temporal organization, predictability, contingency, caregiver buffering, and opportunities for physiological recovery may represent partly separable dimensions of exposure history. Developmental models support considering several of these dimensions but do not establish fixed biological effects for each (Sullivan and Holman, 2010; Del Giudice et al., 2011). Their effects should therefore be tested rather than assumed to combine monotonically or in a predetermined direction.

Successive events are encountered by a system already shaped by prior development. Repetition therefore represents successive inputs to a changing system rather than a fixed dose delivered to an unchanged substrate. Brief developmental perturbations can alter later maturation (Bitzenhofer et al., 2021). More broadly, repeated inputs may recruit sensitization, habituation, compensation, buffering, or recovery depending on the biological state in which they occur; the direction and magnitude of these effects remain empirical questions rather than predetermined consequences of repetition.

This perspective establishes the premise for the mechanistic sections that follow. Early experience does not act on a fixed nervous system and simply leave behind a proportional trace of exposure. Its consequences depend partly on the developmental, endocrine, and circuit conditions under which it occurs, while resulting changes can alter the biological context through which later experience is processed. Developmental timing and prior history are therefore components of the proposed response architecture rather than peripheral modifiers of it.

## 3 Established biological routes of developmental embedding

### 3.1 Canonical HPA, glucocorticoid, and glucocorticoid-receptor signaling

The hypothalamic–pituitary–adrenal (HPA) axis provides one of the clearest established routes through which early experience can influence later stress responsivity. Hypothalamic corticotropin-releasing hormone (CRH) contributes to pituitary activation and downstream glucocorticoid secretion, while glucocorticoid-receptor (GR) signaling participates in negative feedback that limits subsequent HPA activity. GR signaling also regulates transcription, creating a route through which endocrine state can influence longer-term cellular function. Changes in GR abundance or responsiveness can therefore affect both the magnitude of an acute endocrine response and how efficiently that response is brought back down (Meaney and Szyf, 2005; Gunnar and Quevedo, 2007).

Early caregiving can modify this pathway. Weaver et al. (2004) found that naturally occurring variation in maternal licking, grooming, and arched-back nursing in rats was associated with site-specific methylation of the hippocampal Nr3c1 promoter, altered histone acetylation and NGFI-A binding, differences in GR expression, and differences in adult HPA stress responses. Cross-fostering shifted the regulatory pattern toward that of the rearing mother, supporting a developmental relationship between caregiving, chromatin state, GR expression, and stress responsivity. Human postmortem work provides a more limited translational parallel. McGowan et al. (2009) reported increased methylation of the hippocampal exon 1F NR3C1 promoter and reduced GR mRNA in suicide victims with documented childhood abuse. The observational postmortem design supports association rather than direct developmental causality.

Stress-related regulation is not limited to NR3C1. Murgatroyd et al. (2009) found that early-life stress in mice produced persistent hypomethylation of a regulatory region of Avp, sustained AVP overexpression in parvocellular neurons of the paraventricular nucleus (PVN), and long-lasting HPA hyperresponsivity. Klengel et al. (2013) identified a different pattern involving FKBP5 in peripheral blood. In carriers of the rs1360780 risk allele, childhood trauma was associated with allele-dependent demethylation of glucocorticoid-response elements, greater glucocorticoid-induced FKBP5 activity, and reduced efficiency of GR feedback. Because the human methylation measurements were peripheral, they should not be interpreted as direct evidence of the chromatin state of a particular neuronal population. Together, these studies establish multiple endocrine and gene-regulatory routes through which developmental conditions can alter later stress responsivity without implying a single molecular signature of adversity.

### 3.2 Glial contributions to developmental circuit construction

Glial processes provide another route through which early environment can influence the construction of stress-regulatory circuitry. Bolton et al. (2022) found that limited-bedding/nesting adversity during early postnatal development reduced microglial surveillance and engulfment of excitatory synaptic elements adjacent to PVN CRH neurons, produced excess excitatory innervation, and altered later endocrine and behavioral stress responses. Chemogenetic activation of microglia during development normalized synapse density and adult outcomes, providing causal evidence that altered developmental microglial function can contribute to persistent changes in a defined stress-sensitive circuit.

The specificity of this mechanism is important. Peripheral inflammatory findings describe a different level of biological embedding and cannot substitute for direct evidence of microglial pruning or neuronal physiology (Danese and Lewis, 2017; Miller et al., 2011). A peripheral cytokine profile, a monocyte or macrophage phenotype, and microglial synaptic engulfment are related aspects of immune biology, but they are not interchangeable measures. Claims about immune or glial embedding should therefore remain tied to the cell type, tissue, developmental period, and function that were actually measured.

### 3.3 Epigenetic regulation is heterogeneous, not a unitary scar

Epigenetic regulation is best understood here as a family of mechanisms through which developmental history can influence later gene expression. DNA methylation, histone modifications, transcription-factor occupancy, chromatin accessibility, and higher-order chromatin organization are not interchangeable processes, and their consequences depend on genomic location, cell type, developmental stage, and signaling context (Kundakovic and Champagne, 2015). Even within DNA methylation, adversity-related effects do not move in one direction. Weaver et al. (2004), McGowan et al. (2009), Roth et al. (2009), Murgatroyd et al. (2009), and Klengel et al. (2013) identify different loci, tissues, directions, and contingencies. Systematic reviews likewise show substantial heterogeneity across genes, tissues, adversity definitions, developmental periods, and analytic approaches (Jiang et al., 2019; Parade et al., 2021). Tissue is especially important because a methylation difference measured in blood, saliva, placenta, or another peripheral sample cannot be assumed to reproduce the regulatory state of a specific neuronal population (Provençal and Binder, 2015). Nor does the presence of an epigenetic difference by itself establish its physiological consequence. The mechanistically relevant question is how a defined regulatory change alters subsequent cellular function. Section 4 therefore distinguishes the physiological variables that constitute neural electrical response, before Section 5 examines evidence linking neural activity and gene-regulatory state.

## 4 Electrical physiology across intrinsic and synaptic levels

### 4.1 Defining the electrical state of neural systems

Neural electrical state is not a single variable but an interacting set of cellular, synaptic, and population-level properties that determine how incoming signals are transformed into output. In this paper, bioelectrical refers to these electrical properties of neural cells and circuits, including membrane voltage, ion-channel-dependent intrinsic physiology, synaptic drive, and population-level electrical coordination. It does not imply that developmental experience is stored in resting membrane potential (RMP) or any single voltage variable.

At the cellular level, RMP establishes the voltage from which subsequent responses emerge, while ion-channel-dependent intrinsic properties determine how input changes membrane voltage and is converted into action potentials (Zhang and Linden, 2003; Mozzachiodi and Byrne, 2010; Sehgal et al., 2013; Debanne et al., 2019). Input resistance and membrane time constant influence voltage responses and temporal integration; rheobase and spike threshold influence action-potential initiation; and afterhyperpolarization, adaptation, and specific conductances shape subsequent firing. These variables interact but need not change together.

Synaptic physiology describes a distinct but interacting level of neural response. Postsynaptic-current amplitude and frequency, presynaptic release probability, receptor composition, and synaptic strength influence the input reaching a neuron, whereas intrinsic physiology influences how that input is transformed into spike output. Similar firing output can therefore arise through different combinations of synaptic drive and intrinsic gain (Yang and Prescott, 2023). At the population level, these properties contribute to recruitment, synchrony, oscillatory coordination, and functional coupling. The relevant electrical question is therefore not simply whether a neuron is more or less excitable, but which physiological parameters alter input–output transformation. During development, electrical activity also participates in neuronal differentiation and circuit construction through activity-dependent calcium signaling and related developmental programs, functioning both as an information-processing variable and as a developmental signal (Spitzer, 2006).

### 4.2 Stress and developmental perturbations reshape neural electrophysiology

Direct electrophysiological studies indicate that developmental and stress history can alter multiple components of neural input–output function, with affected parameters and direction varying across exposure paradigms, neural populations, developmental stages, sex, and measurement conditions. Changes have been observed in resting voltage, input resistance, ionic conductances, afterhyperpolarization, current-evoked firing, and synaptic transmission, arguing against a unitary state of hyper- or hypoexcitability.

Hu et al. (2020) provide an important direct early-life-stress example. Male C57BL/6 J mice underwent a single 24-h maternal separation beginning at postnatal day 3 and were examined in adulthood. Corticotropin-releasing hormone (CRH) neurons in the oval bed nucleus of the stria terminalis (ovBNST) showed a more depolarized RMP, increased input resistance, reduced KCNQ/Kv7-mediated M-current, and increased miniature excitatory postsynaptic-current (mEPSC) amplitude without a corresponding increase in mEPSC frequency. Reduced M-current weakens a conductance that constrains membrane responsiveness, while increased mEPSC amplitude increases the postsynaptic impact of excitatory input. The phenotype therefore involved interacting intrinsic and synaptic alterations rather than a shifted resting voltage alone.

Hu et al. further linked expression of the adult physiological phenotype to CRH-related signaling. Pharmacological CRHR1 blockade in the ovBNST reversed the maternal-separation-associated reduction in M-current and other physiological and behavioral effects. Exogenous BDNF applied to control tissue reproduced the reduced M-current and increased mEPSC amplitude, whereas PKC antagonism blocked BDNF-dependent effects. These manipulations support an adult contribution of CRHR1-, BDNF-, and kinase-related signaling to expression of the mature phenotype. They do not establish that the same signaling abnormality operated continuously from developmental exposure through adulthood, and the findings remain specific to the exposure paradigm, male mice, and ovBNST CRH neurons.

A different intrinsic configuration is evident following adolescent social isolation. Rau et al. (2015) found that BLA pyramidal neurons from isolated male rats generated more action potentials in response to equivalent depolarizing current even when AMPA- and GABA_A_-receptor-mediated synaptic transmission was blocked. Increased firing was accompanied by reduced medium afterhyperpolarization and reduced SK2/SK3 channel-subunit expression, whereas RMP, input resistance, rheobase, and action-potential threshold were not significantly different. Positive modulation of SK channels reduced the elevated firing and normalized the medium afterhyperpolarization. Because isolation continued until electrophysiological assessment, this study demonstrates a chronic developmental-exposure-associated configuration rather than persistence after the exposure had ended.

Qin et al. (2019) demonstrate a different form of history dependence. Male mice underwent maternal separation for 1 h/day from PND3–21, followed in a subset by chronic social defeat stress in adulthood. Earlier separation attenuated the later stress-induced increases in glutamatergic transmission and intrinsic firing in BLA principal neurons, while several resting and spike-initiation measures, including RMP, threshold, and rheobase, did not change in parallel. Developmental history therefore altered the electrophysiological response to a later challenge without requiring a constitutively abnormal resting value. This finding should not be interpreted as the opposite end of a simple severity continuum with Hu et al.; the studies differ in exposure structure, circuit, and experimental design.

Developmental perturbations outside psychosocial stress models further illustrate parameter specificity. Gomez et al. (2019) reported that inflammatory challenge at P14 produced later sex-dependent changes in dorsal CA1 physiology, including altered current-evoked firing, input resistance, rheobase, spike threshold, and chloride-related physiology while RMP remained unchanged. Because the initiating manipulation was inflammatory rather than psychosocial, this provides developmental mechanistic precedent rather than evidence that psychosocial early-life adversity produces the same hippocampal mechanism. Adult chronic-stress studies likewise demonstrate stress-related ion-channel regulation of amygdala excitability but do not establish developmental programming (Rosenkranz et al., 2010).

Across these studies, electrophysiological variables dissociate in ways that would be obscured by a single measure of “excitability.” RMP can change alongside intrinsic and synaptic properties, firing can increase while RMP and spike-initiation measures remain stable, and developmental history can alter later challenge responsivity without producing an obvious constitutive baseline abnormality. The relevant phenotype is therefore the configuration of parameters that generates neural response, not any one electrical variable considered in isolation.

### 4.3 Intrinsic plasticity as an experience-history mechanism

Neuronal responsiveness is itself plastic. Prior activity can modify membrane conductances so that subsequent inputs are transformed differently even when synaptic strength has not changed (Zhang and Linden, 2003; Mozzachiodi and Byrne, 2010; Sehgal et al., 2013). Alejandre-García et al. (2022) demonstrated this in layer 2/3 pyramidal neurons of mouse visual cortex. Repeated optogenetic or electrical activation increased current-evoked firing, increased membrane resistance, and reduced action-potential threshold while RMP remained stable. Repeated coactivation also increased correlated activity among stimulated neurons, while changes in monosynaptic strength were comparatively modest and previously unconnected pairs did not acquire detectable new connections. Intrinsic plasticity can therefore alter ensemble recruitment without requiring formation of new anatomical connections, consistent with models that distinguish connectivity from excitability-dependent recruitment (Hansel and Yuste, 2024).

Intrinsic plasticity can also operate metaplastically by changing the conditions under which later plasticity or learning occurs. Sehgal et al. (2013) review examples in which prior experience alters afterhyperpolarization, intrinsic excitability, or subsequent learning even when synaptic potentiation follows a different time course. In olfactory rule learning, facilitated acquisition of new odor discriminations overlapped with a temporary increase in intrinsic excitability in piriform cortical neurons; excitability later returned toward baseline while the acquired learning history remained. These findings provide mechanistic precedent for an electrical state influencing how subsequent experience is processed without requiring that state to serve as the permanent storage substrate for the original experience.

### 4.4 Homeostatic regulation, degeneracy, and compensation

Neural systems must remain functional while input, connectivity, growth, and experience-dependent plasticity continually change. Homeostatic regulation can compensate through synaptic scaling, intrinsic conductances, excitation/inhibition adjustments, or combinations of these mechanisms (Davis and Bezprozvanny, 2001; Pozo and Goda, 2010; Turrigiano, 2011, 2012). These mechanisms can alter either incoming synaptic drive or the intrinsic transformation of that drive into firing, with their availability and expression varying across cell type, circuit, developmental stage, and perturbation.

Degeneracy further complicates the relationship between measured output and underlying configuration. Yang and Prescott (2023) define degeneracy as the capacity for structurally different solutions to produce equivalent function. Different combinations of ion-channel expression can therefore yield similar excitability, allowing perturbation of one component to be offset by compensatory changes in others. Pleiotropy creates the complementary problem that altering one conductance can affect multiple cellular properties; preserving one regulated output can therefore coexist with a different underlying physiological configuration.

Similar resting activity or firing consequently does not establish identical underlying physiology, and compensation can preserve one measured output while leaving underlying differences that may become apparent under subsequent perturbation. Conversely, a persistent difference in one parameter does not necessarily imply broad dysfunction if other variables compensate. Baseline and challenge-response measurements should therefore be interpreted together.

### 4.5 A human cellular bridge

Human induced pluripotent stem cell (iPSC)-derived neural models provide a cautious bridge to these mechanisms. Triebelhorn et al. (2024) reported differences in major-depressive-disorder-derived neurons across membrane capacitance, RMP, Na + current density, and spontaneous activity, while measured K + outward-current density did not differ; a greater proportion of neurons also generated spontaneous action potentials when membrane potential was experimentally adjusted to a common depolarized level. The phenotype was therefore distributed across electrical parameters rather than reducible to one excitability measure. However, the neurons were relatively immature, reprogramming can erase or alter epigenetic information, and the model cannot establish childhood adversity as the cause of the observed differences or cleanly separate genetic from retained epigenetic and other influences. Its relevance is therefore as a human cellular precedent for multidimensional electrical phenotypes, not evidence that early adversity itself persisted in those cells.

## 5 Activity-to-genome coupling and state-dependent regulatory updating

### 5.1 Neural activity shapes transcription and chromatin

Neuronal activity can alter gene regulation through converging signaling routes. Synaptic receptor activation and membrane depolarization regulate Ca2 + entry, while the source, localization, amplitude, and duration of Ca2 + signals influence downstream kinase and phosphatase pathways, transcription-factor activity, and chromatin regulation. The genomic response is not a direct readout of membrane voltage or firing rate; electrical and synaptic activity are translated through intracellular signaling pathways that regulate transcription factors such as CREB and AP-1, chromatin accessibility, DNA and histone modifications, and interactions among regulatory elements (Kandel, 2001; Renthal and Nestler, 2008; Sweatt, 2013; Karsten and Baram, 2013; Tognini et al., 2015; West and Greenberg, 2011).

Fernandez-Albert et al. (2019) provide a strong *in vivo* demonstration. In excitatory hippocampal neurons, neuronal activation altered transcription, accessibility of activity-regulated genes and enhancers, transcription-factor occupancy, promoter-enhancer interactions, and higher-order chromatin organization. Much of the transcriptional response was transient, but a subset of AP-1-associated occupancy and regulatory interactions remained detectable after acute activation subsided. Sparse physiological activation during novel-context exploration and the stronger synchronous activation produced by status epilepticus generated overlapping but distinguishable genomic responses. Activity history can therefore modify regulatory state, but the update depends on the pattern and context of activation rather than simply on whether a neuron was active.

Reduced activity can also initiate regulatory change. Korosi et al. (2010) found that augmented neonatal maternal care transiently reduced glutamatergic input onto hypothalamic CRH neurons, including reduced excitatory terminals and spontaneous excitatory postsynaptic-current frequency. Singh-Taylor et al. (2018) subsequently showed that experimentally reducing ionotropic glutamate-receptor activation in hypothalamic tissue reproduced repression of Crh and recruited the transcriptional repressor NRSF/REST, initiating subsequent chromatin changes. Neural activity therefore does not map onto the epigenome through a simple directional rule. Both increased and decreased activity can recruit regulatory programs depending on receptor engagement, signaling context, cell identity, and genomic target. The evidence supports activity-dependent genomic regulation, not a privileged pathway in which a persistent resting membrane voltage directly determines DNA methylation.

### 5.2 Early experience can establish longer-lived regulatory states

Developmental experience can produce regulatory changes that outlast the conditions that initiated them, but their form and persistence are locus-, region-, and exposure-specific. Roth et al. (2009) identified persistent alterations in Bdnf DNA methylation and reduced Bdnf expression in adult prefrontal cortex after infant caregiver maltreatment, while the hippocampal expression pattern differed and was not specific to maltreatment. Murgatroyd et al. (2009) found persistent hypomethylation of an Avp regulatory region after early-life stress, accompanied by persistent Avp upregulation and altered HPA-axis function. Weaver et al. (2004) showed another developmental pathway: maternal-care-associated differences in hippocampal glucocorticoid-receptor promoter methylation emerged during the first postnatal week, followed the rearing mother in cross-fostering, persisted into adulthood, and were experimentally reversible. Together, these studies support persistence of defined regulatory states rather than a generalized increase or decrease in methylation across the brain. Persistence does not imply immutability.

A regulatory consequence also need not require continuous presence of the mechanism that initiated it. In Singh-Taylor et al. (2018), early NRSF and MeCP2 recruitment at Crh did not remain different in adulthood, whereas later repressive histone modifications and altered adult stress responsivity persisted. This temporal dissociation shows that persistence of a regulatory consequence need not require continuous persistence of the initiating molecular state. Persistent epigenetic regulation should therefore be claimed only when temporally separated measurements demonstrate it; an acute transcriptional or chromatin change alone does not establish a durable developmental state.

### 5.3 Regulatory updating is state dependent

The epigenome is not a blank surface onto which experience is copied. A signal reaches a cell whose regulatory architecture already reflects cell identity, developmental stage, available transcription factors, chromatin accessibility, three-dimensional genomic organization, endocrine state, genotype, and previous activity. Fernandez-Albert et al. (2019) showed that different patterns of neuronal activation generate overlapping but distinguishable regulatory responses. Roth et al. (2009) demonstrated region-specific effects within Bdnf regulation, while Klengel et al. (2013) identified a genotype-by-experience interaction in which childhood abuse was associated with FKBP5 demethylation primarily in rs1360780 risk-allele carriers, with greater glucocorticoid-induced FKBP5 activity and altered GR feedback. The regulatory consequence of an experience therefore depends partly on the biological state of the system receiving it.

Kim et al. (2026) provide a particularly direct developmental example. ELS from P10–P17 produced enduring changes in histone modifications in the ventral tegmental area (VTA), including enrichment of H3K4me1, a modification associated with primed or active enhancers, together with increased SETD7. Experimental enrichment of this pathway through juvenile Setd7 overexpression increased H3K4me1 and amplified transcriptional, physiological, and behavioral responses to later adult stress, whereas Setd7 knockdown after ELS reduced later stress sensitivity. Critically, juvenile Setd7 overexpression did not alter the dopamine-neuron input–output curve, action-potential threshold, or input resistance in unstressed adults; increased excitability became evident following adult stress. The manipulation therefore altered later responsiveness rather than simply producing a continuously abnormal electrophysiological baseline.

This challenge dependence distinguishes regulatory priming from constitutive abnormality: a primed state can alter transcriptional or physiological responses to later input even when selected baseline measures do not differ detectably between groups.

### 5.4 Regulatory state can alter intrinsic physiology

The relationship between neural physiology and gene regulation is reciprocal at the level of component mechanisms. Meadows et al. (2016) directly showed that prolonged DNMT inhibition in cultured cortical pyramidal neurons increased current-evoked firing, reduced the medium afterhyperpolarization, and decreased expression of SK-channel-related genes. TET1 knockdown or transcriptional inhibition blocked the excitability increase, supporting a route through cytosine regulation and *de novo* transcription. Importantly, firing increased despite a more hyperpolarized RMP, while several other passive properties were largely unchanged. This experimental dissociation demonstrates that genomic regulation can alter intrinsic input–output function through specific conductance-related mechanisms and that neither RMP nor gene expression alone can stand in for the cell’s full input–output function.

The developmental findings of Kim et al. (2026) provide a complementary *in vivo* example linking regulatory manipulation to later physiological responsiveness. Gene expression, however, is not channel function. Translation, trafficking, membrane localization, subunit composition, phosphorylation, gating, and interacting conductances intervene between transcript abundance and membrane current. Regulatory measurements should therefore be linked to, rather than substituted for, electrophysiological measurements.

### 5.5 Reciprocal coupling across neural activity and gene regulation

The evidence establishes several component relationships with different degrees of directness. Neural activity can alter transcription and chromatin organization, experimental changes in epigenetic regulation can modify intrinsic physiology, and early experience can produce persistent or primed regulatory states that influence later physiology and behavior. Together, these findings support reciprocal coupling between neural activity and gene-regulatory state. What remains untested within a single ELS system is whether an activity-dependent regulatory change can modify physiology in a way that then maintains or regenerates the same regulatory state after the original experience has ended. Reciprocal biological capability is therefore supported, while a self-maintaining reciprocal loop remains an empirical hypothesis.

## 6 Persistence across development: initiation, maintenance, compensation, and substrate handoff

### 6.1 Persistence does not require persistence of the initiating state

A lasting developmental effect can persist after the biological state or process that initiated it has returned toward baseline. A molecular pathway may remain involved in adult expression, a regulatory state may preserve altered responsiveness, or a transient developmental perturbation may redirect circuit construction. Continued environmental conditions can also repeatedly recruit an established trajectory without requiring one autonomous biological mechanism to maintain it (Blair and Raver, 2012; Miller et al., 2011). Persistence of a developmental consequence therefore does not require one abnormal variable to remain continuously altered from infancy to adulthood.

The term substrate handoff refers specifically to a change across development in the mechanism carrying the functional consequence of an earlier event. The Korosi/Singh-Taylor sequence illustrates a pattern consistent with substrate handoff: an early reduction in excitatory input to PVN CRH neurons dissipated while downstream regulatory changes and altered later stress responsivity persisted (Korosi et al., 2010; Singh-Taylor et al., 2018). The adult phenotype therefore need not represent a frozen version of the neonatal state.

This distinction separates initiation, maintenance, and expression. A factor can be necessary for establishing a developmental change but unnecessary once structural or regulatory consequences have formed. Conversely, a pathway can contribute to adult expression without having been continuously active throughout development. Demonstrating substrate handoff requires temporally separated evidence linking the candidate substrates. An early variable should change during or soon after the perturbation and later normalize or lose explanatory power, while a mechanistically connected substrate emerges or persists and becomes predictive or causal for mature function. Without that temporal and mechanistic connection, different abnormalities observed at different ages are not sufficient to establish handoff.

### 6.2 Distinct mechanisms can carry developmental history across time

Developmental effects can persist through several nonexclusive mechanisms, and substrate handoff is only one possibility. Continued pathway involvement is illustrated by the contribution of CRHR1-related signaling to the mature physiological phenotype described by Hu et al. (2020), although continuous involvement from infancy through adulthood was not demonstrated. Developmental circuit remodeling provides another route, as early microglial or activity perturbations can alter later circuit organization (Bolton et al., 2022; Bitzenhofer et al., 2021). Regulatory priming provides another route by which developmental history can influence later function without requiring a continuously abnormal adult baseline (Kim et al., 2026). In other cases, a developmental process may give way to a different mechanistically connected substrate that carries its functional consequence forward (Figure 2).

These persistence modes can coexist. Early synaptic change, regulatory stabilization, structural reorganization, continued pathway involvement, compensation, and later environmental input may carry different explanatory weight at different developmental stages (Yang and Prescott, 2023). The mechanistic question is therefore not whether one abnormal variable can be followed unchanged from infancy to adulthood, but which substrate best explains function at each developmental stage and under which conditions that developmental history becomes visible.

Distinguishing among continuous persistence, latent priming, structural reorganization, compensation, environmental maintenance, and substrate handoff requires temporally resolved designs that measure candidate mechanisms at multiple developmental stages. Where relevant, measuring both baseline and challenge-evoked function can further determine whether an apparently normalized system responds differently when recruited. Such designs can establish whether the same substrate remains functionally important across development or whether the biological mechanism carrying the functional consequences of earlier experience has changed.

## 7 Scaling from cellular state to circuits and later behavior

### 7.1 Intrinsic state can bias ensemble recruitment

Changes in intrinsic physiology can influence circuit function by altering the probability that a neuron will be recruited by a given synaptic input (Zhang and Linden, 2003). As established in Section 4.3, activity-dependent changes in membrane resistance and spike threshold can change the effectiveness of existing input without requiring new anatomical connections (Alejandre-García et al., 2022). At the population level, such differences can alter which neurons participate in a response.

This principle is consistent with the engram-allocation literature. Relative excitability can bias which neurons are recruited into memory ensembles, while synaptic and structural changes and distributed interregional connectivity still contribute to storage and retrieval (Josselyn and Tonegawa, 2020; Sweatt, 2016). Intrinsic excitability is therefore a recruitment bias rather than a complete memory mechanism. A coactive ensemble also need not automatically be labeled an attractor; that stronger claim requires state-space and perturbation evidence considered in Section 10.

Altered recruitability can change the effective influence of existing connectivity. Anatomically similar circuits may recruit different neuronal populations when the cells receiving those projections differ in intrinsic response properties. Cellular physiology can therefore influence ensemble composition without requiring intrinsic excitability itself to serve as the complete storage mechanism.

### 7.2 Developmental activity can redirect network maturation

A cellular change acquires longer-term significance when it occurs while activity is helping construct the circuit. Bitzenhofer et al. (2021) transiently increased coordinated activity of medial prefrontal layer 2/3 pyramidal neurons from P7–P11. The manipulation produced premature pyramidal-cell maturation and altered interneuron development, followed in adulthood by altered inhibitory feedback, E/I organization, weaker evoked gamma synchronization, and cognitive and social deficits. The initiating perturbation was temporary, but its consequences were expressed through mature circuit organization.

Importantly, the adult difference was not equally visible under all conditions. Spontaneous fast oscillatory activity was relatively similar at later stages, whereas gamma power was reduced during social interaction and after optogenetic network recruitment. The study therefore demonstrates a response-function difference more clearly than a continuously expressed resting-state abnormality. Bitzenhofer et al. is not an ELS experiment; its importance here is causal demonstration that transient developmental activity can redirect later circuit maturation.

Developmental circuit organization can also be altered through non-neuronal mechanisms, including microglia-dependent synaptic remodeling during ELS (Bolton et al., 2022). Intrinsic recruitment bias, synaptic remodeling, and glial pruning are complementary mechanisms rather than mandatory stages of a single cascade. Developmental experience can therefore influence mature circuit operation through multiple routes without requiring every mechanism to be altered in every system.

### 7.3 Network differences can emerge under recruitment

Mature circuit differences need not be equally apparent during spontaneous and recruited states. As the Bitzenhofer et al. (2021) findings illustrate, relatively similar spontaneous activity can coexist with altered synchronization when a network is engaged by behavior or experimental stimulation. A developmental perturbation can therefore alter the conditions under which a circuit coordinates its activity without producing a continuously abnormal resting output.

At a broader translational level, this recruitment-dependent logic is compatible with latent-vulnerability models, in which maltreatment-associated neurocognitive alterations may precede overt psychiatric disorder and become more consequential under later developmental or environmental challenge (McCrory and Viding, 2015; McCrory et al., 2017). These human findings do not identify the cellular mechanism proposed here, but they support the broader distinction between latent developmental alteration and continuously expressed dysfunction.

Challenge-evoked measurements may therefore reveal circuit differences that are not apparent at rest. Extending such designs to the trajectory of recovery after recruitment would test whether developmental history also alters how the system returns toward baseline. The relevant network phenotype may therefore include how strongly a circuit is recruited, which populations participate, how activity is coordinated, and potentially how the system returns toward baseline after recruitment.

## 8 The integrated theory: a history-dependent response architecture

The mechanisms described in the preceding sections suggest that developmental experience need not be stored in any single persistent biological variable. Neural and stress-regulatory systems instead comprise interacting intrinsic physiological, synaptic, transcriptional and epigenetic, endocrine, immune and glial, and network processes. Similar functional outputs can arise from biologically distinct underlying configurations, including through compensatory and degenerate mechanisms. Different combinations of ion-channel properties, for example, can produce similar functional outputs (Yang and Prescott, 2023). Functionally similar systems therefore need not be biologically identical, and restoration of an observable function does not necessarily imply restoration of the configuration that previously generated it (Figure 3).

Developmental experience can alter this architecture, after which subsequent experience acts on the system produced by what came before. Developmental history is therefore iterative rather than simply cumulative: an event can alter the conditions under which the next event is encountered. Successive inputs may therefore reinforce, attenuate, or redirect an existing trajectory rather than simply adding to it. The consequences of a perturbation depend partly on developmental timing because the regulatory, cellular, and circuit processes available during maturation are themselves changing. Transient manipulation of neonatal prefrontal activity during circuit formation, for example, produced later alterations in excitation/inhibition organization, gamma synchronization, and behavior that depended on the developmental window of manipulation (Bitzenhofer et al., 2021). Developmental history is therefore better represented as a temporally structured sequence than as cumulative dose alone. Predictability, contingency, spacing, clustering, opportunities for recovery, and their interactions may contribute to that temporal organization, but their effects remain empirical questions rather than having predetermined beneficial or harmful directions (Sullivan and Holman, 2010; Del Giudice et al., 2011).

Persistence of developmental influence likewise does not require persistence of the same biological alteration. Transient developmental perturbation can produce later changes after the initiating manipulation has ended (Bitzenhofer et al., 2021), while early experience can produce persistent, site-specific epigenetic differences whose functional consequences depend on genomic and regulatory context (Roth et al., 2009; Provençal and Binder, 2015). Developmental consequences may persist through continued pathway involvement, latent regulatory priming, structural reorganization, changing substrates, or continued environmental conditions. The biological feature most informative shortly after an experience therefore need not be the feature most informative later in development, and apparent normalization at one level does not necessarily indicate restoration of the earlier biological configuration.

A central implication is that developmental history may alter how a system responds when subsequently recruited even when resting measures overlap. Developmental prefrontal perturbation can leave evoked network synchronization altered despite relatively similar spontaneous activity (Bitzenhofer et al., 2021), while developmental regulatory priming can amplify later stress responses despite limited differences in selected baseline electrophysiological measures (Kim et al., 2026). Developmental history may therefore be expressed more clearly during recruitment or challenge than under selected baseline conditions. The enduring difference may lie partly in input-response relationships involving threshold, gain, recruitment, synchronization, or adaptation, while altered recovery dynamics remain an additional testable possibility. Similarity under one resting condition cannot by itself establish equivalence of the biological systems generating that output.

The multidimensional character of the architecture also means that developmental history may alter relationships among biological levels rather than producing an isolated abnormality in each one. Intrinsic physiology, synaptic organization, regulatory state, endocrine signaling, glial processes, and network recruitment need not vary independently, and compensation at one level may alter how differences at another are expressed. One empirical question is whether developmental groups instead differ in how these levels covary or jointly predict response during recruitment. This possibility is an inference of the integrated framework rather than a relationship established across all mechanisms reviewed here.

The framework consequently predicts heterogeneity rather than a universal direction of biological change. Similar cumulative exposure need not produce identical mature architectures when temporal organization or biological context differs, while different underlying architectures may sometimes converge on similar observable outputs. These propositions extend beyond what any single study reviewed here demonstrates and should therefore be treated as predictions of the integrated framework rather than universal mechanisms. Section 9 considers the moderators and forms of this heterogeneity in greater detail.

A direct test would compare developmental histories matched approximately for cumulative exposure but differing in temporal organization. If dimensions such as spacing, clustering, predictability, contingency, or recovery predict later baseline physiology or challenge-evoked response dynamics beyond cumulative exposure, this would support a history-dependent architecture over a simpler cumulative-exposure model. The direction of these effects should not be assumed in advance and may itself depend on developmental stage and biological context.

The framework adds explanatory value only where this history-dependent architecture accounts for findings better than a simpler persistent-variable explanation. It predicts that developmental groups may differ in challenge sensitivity, response dynamics, compensation, or relationships among biological levels despite overlapping baseline measures. Conversely, if a single enduring mechanism explains later responses across developmental stages, contexts, and challenges equally well, the simpler account should be preferred. The central claim is therefore not that developmental adversity produces a universal biological state, but that developmental conditions can alter the biological architecture through which subsequent inputs are transformed into physiological and behavioral responses.

## 9 Heterogeneity is part of the mechanism, not noise

### 9.1 Exposure dimensions

Heterogeneity begins with the structure of the developmental experience itself. Experiences grouped under early-life stress differ in developmental timing, physical intensity, duration, repetition, caregiver involvement, predictability, contingency, and opportunities for recovery. Maternal separation, limited-bedding caregiving, neonatal inflammation, social isolation, and direct threat therefore cannot be assumed to constitute interchangeable quantities of a common “stress dose.” They engage different combinations of sensory, endocrine, neural, immune, and social processes during different periods of development (Del Giudice et al., 2011; Blair and Raver, 2012; Tottenham and Sheridan, 2010; Teicher and Samson, 2016).

Within a history-dependent framework, these differences are mechanistically relevant rather than merely sources of experimental variability. Two developmental histories with similar cumulative exposure may differ in which biological systems are repeatedly recruited, the developmental state in which recruitment occurs, and the physiological conditions established before subsequent events are encountered. Exposure type and temporal organization should therefore be treated as dimensions capable of contributing to different developmental trajectories rather than collapsed into a single cumulative severity measure. The specific contribution of each dimension remains an empirical question; the framework does not assign fixed biological consequences to spacing, clustering, predictability, contingency, or any other temporal feature in advance.

This distinction is also necessary when integrating evidence across experimental models. Mechanistic findings from different early-life adversity paradigms can identify shared developmental capabilities or principles without implying that the exposures themselves are biologically or phenomenologically equivalent. Heterogeneity across paradigms should therefore be preserved when constructing the framework rather than averaged into a single generalized effect of “early-life stress.”

### 9.2 Sex as a developmental moderator

Sex should likewise be treated as a developmental moderator rather than as a uniform directional effect. The relevant question is not whether males or females are globally more vulnerable, but whether sex changes the timing, magnitude, or biological route through which a particular exposure affects a particular cell population or circuit (Provençal and Binder, 2015; Teicher and Samson, 2016).

Bolton et al. (2022) provide a direct example. In their limited-bedding/nesting model, males showed increased excitatory synapses onto PVN CRH neurons and reduced microglial synapse engulfment during the early adversity period. Females did not show the same neonatal synaptic and pruning phenotype, but a more modest increase in CRH-neuron excitatory synapses emerged later. Subsequent mechanistic and rescue experiments were conducted primarily in males. The important distinction is therefore not simply one of effect magnitude; the measured developmental trajectory itself differed by sex (Bolton et al., 2022).

Gomez et al. (2019) likewise found delayed intrinsic physiological effects of neonatal inflammation in adult males but not females. Because the initiating manipulation was neonatal inflammation rather than psychosocial adversity, this provides a developmental physiological precedent rather than direct evidence about human trauma (Gomez et al., 2019). These findings argue against assuming that findings from one sex generalize automatically to the other.

### 9.3 Region, cell type, and microanatomy

A generalized brain-wide electrical phenotype is too coarse to describe the evidence reviewed in Sections 3–7. Developmental effects have been identified in defined regions and populations, including ovBNST CRH neurons, BLA and hippocampal pyramidal neurons, VTA dopamine neurons, prefrontal populations, and hypothalamic stress-regulatory circuitry (Hu et al., 2020; Rau et al., 2015; Gomez et al., 2019; Bolton et al., 2022; Bitzenhofer et al., 2021; Kim et al., 2026). Their differing physiological configurations cannot be collapsed into a general claim that developmental adversity simply increases or decreases neural excitability throughout the brain.

Specificity can extend below the level of the anatomical region. Bolton et al. (2022) identified increased excitatory synaptic density on CRH-expressing neurons in the mediodorsal parvocellular PVN, whereas a nearby hypothalamic region not enriched for CRH neurons did not show the same synaptic difference. Spontaneous excitatory current frequency increased without parallel changes in amplitude or inhibitory spontaneous currents (Bolton et al., 2022). The findings support a microglia-to-excitatory-synapse mechanism in a defined stress-regulatory population rather than a generalized alteration in hypothalamic excitability.

Cell identity further changes the interpretation of a physiological alteration. Glutamatergic pyramidal neurons, GABAergic interneurons, CRH-expressing neuroendocrine neurons, and dopamine neurons occupy different functional positions within their circuits. The same nominal physiological change therefore need not have the same network consequence across populations (Hu et al., 2020; Bolton et al., 2022; Kim et al., 2026). The proposed architecture must refer to defined biological systems rather than treating the brain as a homogeneous electrical substrate.

### 9.4 Genotype, prior regulatory state, and Equifinality

The biological consequences of an exposure also depend on the system that receives it. Klengel et al. (2013) found that the relationship between childhood abuse and FKBP5 regulation depended on rs1360780 genotype. Childhood abuse was associated with demethylation of glucocorticoid-response-element-associated CpGs within FKBP5 intron 7 primarily in carriers of the risk allele. The risk allele was also associated with altered FKBP5 chromatin conformation and stronger glucocorticoid-responsive transcription, while increased FKBP5 induction has been linked to reduced efficiency of glucocorticoid-receptor feedback (Klengel et al., 2013). The same broad environmental category therefore did not produce the same regulatory association across genetic backgrounds.

Genotype is only one component of the receiving context. As developed in Section 5, existing regulatory and physiological state can likewise influence how later signals are processed (Fernandez-Albert et al., 2019; Kim et al., 2026). Prior state can therefore change what a later input is capable of doing.

This creates the possibility of both multifinality and equifinality. Broadly similar developmental experiences can lead to different mature response architectures when the receiving systems differ, while similar observable outcomes can emerge through different underlying biological routes. Similar symptoms therefore need not imply identical combinations of intrinsic physiology, synaptic organization, endocrine regulation, network recruitment, or regulatory priming, and neither a particular symptom nor a single physiological or molecular measurement uniquely identifies the developmental pathway that produced it.

### 9.5 Resilience and compensation

Heterogeneity also includes developmental trajectories in which later stress responsivity is attenuated rather than amplified. Singh-Taylor et al. (2018) provide an example arising from augmented maternal care: adults exposed to this early-life condition showed reduced corticosterone responses to acute restraint stress without altered basal corticosterone, together with reduced anxiety-like behavior and behavioral measures interpreted as resilience to depressive-like behavior. These adult outcomes accompanied enduring repression of hypothalamic Crh and persistent repressive chromatin modifications established following earlier changes in excitatory input and NRSF/MeCP2 recruitment (Singh-Taylor et al., 2018). The findings therefore demonstrate that developmental biological embedding can support a comparatively buffered later stress phenotype rather than necessarily producing heightened responsivity.

Qin et al. (2019) provide a distinct form of attenuated later responsivity within an adversity paradigm. Male mice exposed to maternal separation from PND3–21 subsequently showed attenuation of social-defeat-associated anxiety-like behavior and of the accompanying increases in glutamatergic transmission and intrinsic excitability in BLA principal neurons. As discussed in Sections 2 and 4, this result does not establish maternal separation as generally protective or imply that increasing developmental adversity produces increasing resilience. Rather, it demonstrates that an earlier developmental history can modify the direction and magnitude of physiological and behavioral responses to a later challenge.

The functional meaning of such adaptations remains context dependent. Developmental-calibration models distinguish biological adaptation from psychological well-being and allow a configuration shaped under one set of conditions to carry different consequences when later environmental demands change (Del Giudice et al., 2011). Resilience should therefore be understood here operationally as maintenance or recovery of specified functions under specified challenges, not as the absence of biological adaptation, a universally beneficial phenotype, or a fixed property of the individual.

The heterogeneity reviewed here is demonstrated most directly across experimental paradigms, developmental stages, sexes, regions, cell populations, and measurement conditions. Extending these differences to individualized mechanistic architectures in humans remains a prediction of the framework rather than a conclusion established by the present evidence. The stronger conclusion is that developmental adversity cannot presently be treated as producing one uniform biological phenotype.

## 10 Bioelectrical regulation across neural and developmental scales

### 10.1 Developmental bioelectricity as a bounded cross-domain precedent

Developmental and regenerative biology provide a broader precedent for the possibility that bioelectrical variables can participate instructively in developmental regulation rather than functioning only as downstream correlates of cellular activity. In these systems, membrane voltage, ion-channel activity, and intercellular electrical communication interact with biochemical and gene-regulatory processes involved in development, patterning, and regeneration (Chernet and Levin, 2013; Cervera et al., 2020; Pietak and Levin, 2017; Whited and Levin, 2019).

Experimental manipulation of membrane potential has altered regenerative patterning in planaria (Beane et al., 2013; Durant et al., 2017), while Adams et al. (2013) used optogenetic activation of a proton pump in Xenopus to alter regenerative capacity. Importantly for the present framework, endogenous resting-potential gradients have also been shown to instructively pattern embryonic neural tissue through Notch signaling and regulation of proliferation (Pai et al., 2015). These findings establish that bioelectrical variables can participate in developmental regulation across multiple biological contexts, but they do not demonstrate that an experimentally altered voltage state must itself persist as the substrate of a later developmental consequence (Durant et al., 2019).

This literature is used here as cross-domain evidence for a biological capability, not as evidence that the developing human brain stores trauma as a morphogenetic voltage map. Regenerative tissues, embryonic neural tissue, and mammalian stress-responsive neural circuits differ substantially in organization and function. Whether comparable bioelectrical principles contribute to developmental embedding within a particular neural circuit must therefore be demonstrated within that system.

### 10.2 Attractor dynamics as a testable extension

Persistence alone is not sufficient to establish an attractor. An attractor account requires a defined state space and evidence that system trajectories exhibit characteristic stability following perturbation. Vinograd et al. (2024) provide a neural benchmark: population activity of Esr1-expressing VMHvl neurons was modeled as an approximate line attractor, and targeted optogenetic perturbations tested predicted movement and recovery within that state space. Perturbation along the modeled integration dimension moved population activity along the attractor, whereas displacement away from the manifold was followed by relaxation toward it. This combination of state-space identification, causal perturbation, and recovery provides a stronger standard for attractor evidence than persistence or recurrent activity alone.

Vinograd et al. (2024) do not establish that early-life stress creates, shifts, or stabilizes an attractor, and no source in the present evidence base demonstrates that developmental adversity changes an attractor’s location, basin, stability, coupling, or return dynamics. Attractor reconfiguration is therefore a testable extension of the history-dependent architecture rather than a premise of it. If developmental history alters stable network dynamics, exposed and comparison groups should differ in measurable state-space trajectories following controlled perturbation, including displacement, recovery, transition thresholds, or stability. If persistent developmental differences occur without corresponding differences in perturbation-response dynamics, the stronger attractor interpretation would not be supported while the broader history-dependent architecture could remain intact.

Developmental bioelectricity and attractor dynamics therefore provide bounded extensions of the framework rather than evidence that preverbal trauma is stored as a persistent voltage pattern or attractor state. The central claim remains narrower: developmental experience can alter interacting physiological and regulatory processes that influence how the system responds to subsequent conditions.

## 11 Experimental program: mapping a history-dependent response architecture

### 11.1 Core design principle: developmental history × later challenge × recovery

A core test should place developmental history and later challenge within the same experiment. A prechallenge measure establishes the starting state, the evoked response shows how the system is recruited, and response magnitude, gain, attenuation, and recovery characterize the trajectory that follows. Bitzenhofer et al. (2021) and Kim et al. (2026) provide complementary precedents in which consequences of developmental history became especially apparent when mature systems were subsequently engaged.

Sampling during or shortly after exposure, at an intermediate developmental period, and after maturation can establish when a candidate substrate first appears and whether the variable most informative of the phenotype changes across development. Including both sexes allows sex-specific trajectories to be identified rather than treating sex solely as a *post hoc* covariate, while prespecifying the target region and cell population constrains mechanistic inference. When temporal structure is tested, its dimensions should be manipulated independently of cumulative exposure where biologically feasible.

### 11.2 Comparative olfactory development as a tractable testbed

The infant rat olfactory attachment system described in Section 2 provides a tractable testbed because developmental stage, maternal presence, corticosterone state, sensory contingency, and learned preference versus avoidance can be manipulated or measured across a defined developmental transition (Moriceau and Sullivan, 2006; Sullivan and Holman, 2010). One core design could compare attachment-sensitive and transitional stages while varying maternal presence during a defined odor-conditioning experience. A caregiving-history manipulation could then test whether earlier developmental conditions alter the endocrine and circuit processes governing recruitment of preference-versus-avoidance learning. As established in Section 2, this is a comparative developmental model rather than a reproduction of human preverbal trauma.

Temporal structure can be introduced as a second experimental axis by varying contingency, spacing, clustering, or recovery opportunity while holding event number and approximate physical intensity as comparable as the biology permits. Paired and unpaired odor-shock exposure provide one biologically coherent contrast because contingency during infant odor-shock exposure has been associated with divergent later behavioral outcomes (Sullivan and Holman, 2010). Importantly, the available evidence does not establish a universal effect of spacing, clustering, or recovery duration; these should therefore be manipulated as experimental variables rather than assigned predetermined effects. Recovery should be measured physiologically rather than inferred from elapsed time alone. This design would test whether the organization of repeated experience contributes information beyond cumulative exposure.

After maturation, developmental-history groups can be exposed to the same later challenge. Analysis can then determine whether early history alters prechallenge state, evoked magnitude, gain, recruitment, adaptation, or recovery. Measurement should follow the causal hypothesis. Electrophysiology should distinguish resting, intrinsic, and synaptic components rather than treating excitability as a unitary measure. Endocrine measurements can capture baseline, peak response, and recovery; molecular or chromatin assays can test proposed regulatory bridges; and network measures can quantify recruitment, coupling, excitation-inhibition organization, and evoked synchronization where the preparation permits.

The same principle applies to molecular targets. GR/NR3C1, BDNF-related signaling, ion-channel transcription, SETD7/H3K4me1, or another regulatory measure becomes informative when a specific relationship between that measure and the physiological phenotype is proposed. Including a molecule only because it is commonly associated with adversity adds little. A stronger experiment asks whether the molecular measure predicts cellular or circuit function and whether perturbing the proposed link changes the later response.

Because electrophysiological, histological, and genomic assays are frequently destructive, matched developmental cohorts may be more practical than repeated measurement of the same animal. Early, intermediate, and adult cohorts could, respectively, characterize the initial response, emerging regulatory or structural changes, and mature challenge responsivity.

### 11.3 Model systems should answer distinct mechanistic questions

No single preparation can test every component of the framework. Models should instead be selected for the mechanistic question they are suited to isolate, including developmental circuit construction, persistent intrinsic or synaptic physiology, regulatory priming, and activity-dependent network maturation. Convergence across preparations is therefore more informative than forcing biologically distinct models into one universal representation of preverbal trauma.

A modular experimental program also reduces the risk of false convergence. Stronger support would come from principles such as challenge-revealed responsivity or temporal substrate handoff appearing across preparations through mechanisms appropriate to each system, rather than requiring an identical biomarker across ovBNST, PVN, VTA, hippocampal, and prefrontal circuits.

### 11.4 Causal tests, substrate handoff, and falsification

The timing of a causal manipulation determines the inference it supports. Manipulation during exposure can test initiation; manipulation after exposure but before maturation can test consolidation or maintenance; adult manipulation can test expression of the mature phenotype; and manipulation during or after challenge can test response regulation or recovery. Consequently, reversal of an adult physiological phenotype identifies a pathway contributing to its current expression but does not by itself establish that the same pathway initiated the developmental change. Conversely, preventing a phenotype through developmental manipulation can establish a role in initiation or circuit construction without implying that the initiating process remains abnormal in adulthood. Keeping these causal roles separate is necessary for distinguishing continuous persistence from substrate handoff.

A direct test of substrate handoff requires measurements across several developmental positions. The strongest pattern would show an initiating variable declining or normalizing while a mechanistically connected downstream substrate persists, followed by evidence that manipulating the later substrate changes mature responsivity. If adult effects are fully explained by uninterrupted persistence of the initiating variable, the simpler persistence account is sufficient.

The framework would be weakened if developmental history produced no reproducible alteration in later input–output function once current context was controlled or if proposed regulatory differences failed to predict or causally influence later physiology. Claims concerning temporal organization would likewise require narrowing if manipulating temporal structure produced no reproducible difference after event number and approximate intensity were controlled.

Specificity provides an additional test. If different challenge domains produce the same group difference regardless of the circuits recruited, the model may be detecting a broad state variable rather than the proposed region- and mechanism-specific response architecture. If differences instead emerge preferentially when an altered circuit is recruited, the result would support a more specific response-function account. Testing more than one later challenge, where feasible, can therefore help distinguish generalized sensitization from domain-specific reconfiguration.

### 11.5 Human translation across measurable levels of analysis

Human studies can test systems-level consequences of developmental history while keeping inference at the level actually measured. Relevant outcomes include task-evoked threat, reward, or regulatory processing; endocrine and autonomic challenge trajectories; recovery following stress; longitudinal symptom emergence; and interactions with later protective or adverse environments. Prospective designs are especially informative when a response profile predicts later vulnerability among individuals with similar exposure histories rather than merely distinguishing exposed from unexposed groups.

A narrower sensory example comes from Zheng et al. (2024), who reported associations among childhood-trauma history, olfactory dysfunction, and internalizing problems in young adults, with olfactory dysfunction statistically mediating part of the relationship between childhood trauma and internalizing problems (Zheng et al., 2024). This provides a correlational human bridge, not evidence that the infant-rodent olfactory mechanism operates in humans.

Human translation should preserve the experimental logic of the animal work without assuming biological equivalence across measurement scales. The relevant approach is to follow response trajectories, identify the conditions under which differences become apparent, and test prospectively whether those profiles predict later vulnerability. A BOLD, autonomic, or endocrine phenotype therefore need not be presented as the human equivalent of a particular rodent cellular mechanism.

Measurement boundaries should remain explicit. BOLD activation is not neuronal resting membrane potential, EEG is not intrinsic excitability, peripheral methylation is not neuronal chromatin state, and peripheral inflammatory markers are not measures of microglial synaptic pruning. Animal and human evidence can converge on the principle of history-dependent responsivity without requiring identical circuitry, molecular substrates, or phenomenology.

## 12 Translational significance: toward mechanism-informed phenotyping

The translational implication of the framework is to separate exposure history, latent vulnerability, current symptoms or functional state, and causal biological mechanism as distinct targets. A measure associated with one cannot automatically identify the others. McCrory and Viding (2015) define latent vulnerability as a measurable alteration that can precede overt psychopathology, and subsequent neuroimaging work identifies maltreatment-associated differences across threat, reward, emotion-regulation, and executive-control systems even in some young people who do not currently meet criteria for disorder (McCrory et al., 2017). If the present framework proves translationally useful, a mechanistic phenotype would therefore describe a response function rather than one abnormal resting biomarker. Relevant dimensions could include resting value, activation threshold, response gain or slope, peak magnitude, adaptation, recovery, context sensitivity, and coordination among neural, endocrine, autonomic, and behavioral systems (Del Giudice et al., 2011).

This approach also follows from equifinality and multifinality. Similar symptoms can arise through different combinations of threat-circuit recruitment, endocrine responsivity, synaptic organization, intrinsic physiology, or regulatory priming, while similar developmental histories can lead to different response profiles depending on biological moderators and later context. Exposure category or symptom category therefore should not be assumed to identify a unique underlying mechanism. The longer-term goal would be to determine whether validated response profiles can distinguish mechanistically meaningful forms of vulnerability among individuals who appear similar by exposure or symptoms alone. Mechanism-informed phenotyping would add another level of description to symptom-based diagnosis rather than simply divide existing diagnoses into smaller groups.

Validated response profiles could eventually contribute to prospective risk stratification, enrollment in mechanistic studies, selection of biological targets, or treatment matching, but these applications would require longitudinal validation, reliability, specificity, and evidence that the profiles predict clinically meaningful outcomes beyond simpler measures. At present, the framework is a mechanistic research model rather than a diagnostic or treatment-selection tool. It cannot infer an individual’s trauma history from a biological profile. Its immediate translational contribution is therefore narrower: to identify response dimensions and mechanistic levels that can be tested prospectively before stronger diagnostic, prognostic, or treatment-related applications are considered.

## 13 Limitations

The principal limitation of the present framework is that its integrated architecture has not been demonstrated end-to-end within a single experimental system. The evidence reviewed here establishes individual relationships among developmental experience, stress physiology, intrinsic and synaptic function, activity-dependent gene regulation, chromatin state, glial processes, and network development across different species, preparations, developmental periods, brain regions, and cell populations. Integrating these findings into a history-dependent response architecture is therefore a theoretical synthesis rather than evidence that all of these mechanisms occur sequentially, simultaneously, or in every individual exposed to early adversity. Evidence supporting particular links should not be interpreted as direct demonstration of the complete proposed architecture.

The strength of evidence is also uneven across components of the framework. Direct developmental-stress evidence for persistent changes in resting membrane potential is substantially narrower than evidence for changes in intrinsic excitability, synaptic drive, ion-channel-dependent physiology, activity-dependent regulation, and circuit organization. Likewise, activity-dependent chromatin remodeling establishes a mechanism through which neural activity can influence gene regulation, but persistence and causal relevance of any particular epigenetic state must be demonstrated in the relevant developmental model rather than assumed. Evidence from adult or adolescent stress preparations can establish mechanistic capability but cannot be treated as direct evidence for preverbal early-life stress. More generally, measurements at different biological levels are not interchangeable: channel expression does not establish channel function, and synaptic drive is not intrinsic excitability.

Translation from experimental models to human preverbal trauma requires similar restraint. Animal early-life stress paradigms allow causal isolation of developmental processes but do not reproduce the subjective phenomenology of human trauma, and human neuroimaging or peripheral biomarkers do not provide direct access to the cellular mechanisms measured experimentally in animal preparations. Developmental bioelectric patterning in regenerative and morphogenetic systems therefore serves only as cross-domain evidence that electrical variables can participate instructively in biological regulation; it does not demonstrate that psychological trauma is stored as a tissue-level voltage pattern. Likewise, demonstration of continuous-attractor dynamics in another mammalian neural circuit establishes that such dynamics can be measured and causally tested, not that early-life stress creates or modifies a neural attractor.

Finally, the framework does not predict a single biological trajectory or biomarker of preverbal trauma. Developmental stage, sex, genotype, exposure characteristics, neural region and cell population, later environment, and other biological context can alter the direction, magnitude, persistence, or functional significance of developmental effects. Temporal features such as spacing, clustering, predictability, contingency, and recovery are proposed as experimentally separable dimensions of developmental history, but the current evidence does not establish a universal temporal pattern that is protective or harmful across systems. The framework should therefore be understood as a mechanistic hypothesis and measurement program for testing history-dependent response architecture, not as a diagnostic signature, a means of inferring an individual’s trauma history, or a current treatment-selection framework.

## 14 Conclusion

Preverbal trauma occurs while neural, endocrine, and regulatory systems involved in stress, emotion, learning, and physiological response are still developing. Early developmental experience can therefore remain biologically consequential even when later autobiographical or linguistic access to the original experience is limited. Limited narrative access does not imply an absence of early learning or biological influence, but neither should biological embedding be equated with autobiographical memory. Developmental conditions can instead influence the maturation of systems that subsequently regulate how the organism responds to its environment.

This distinction between persistence of influence and persistence of state is central to the proposed framework. A developmental perturbation may alter processes that subsequently participate in circuit maturation, regulatory development, or later plasticity, allowing its consequences to persist even if the initiating alteration diminishes or normalizes. In other cases, a molecular, cellular, or circuit alteration may remain relatively stable, or a latent difference may become apparent primarily when the mature system is challenged. These possibilities are not mutually exclusive. Subsequent experience acts on an organism already shaped by prior conditions, allowing developmental history to remain influential through the trajectory it helped establish.

The framework conceptualizes this developmental consequence as a multidimensional, history-dependent response architecture. Bioelectrical organization is one interacting component of this architecture rather than a synonym for persistent alteration in resting membrane potential. Intrinsic physiology, synaptic organization, regulatory state, endocrine and glial processes, and network organization may contribute at different developmental periods and timescales, while compensation and subsequent development may alter which processes most strongly express the influence of earlier experience. Developmental history may therefore become apparent through how a system responds when recruited rather than through a continuously abnormal resting measure. Similar resting or behavioral outputs can coexist with differences in response gain, recruitment, adaptation, coordination, or recovery, while broadly similar developmental exposures can produce different outcomes depending on timing and biological context. The central proposal is therefore not that preverbal trauma produces one enduring abnormal state, but that developmental experience can alter the evolving biological architecture through which subsequent inputs are transformed into physiological and behavioral responses.

The value of this framework ultimately depends on whether these propositions survive experimental testing. Rather than asking only whether an exposed group differs from a comparison group at rest, the framework directs attention to when developmental differences emerge, which biological processes carry those differences across development, and under what conditions they become functionally apparent. Longitudinal or matched-cohort designs that combine developmental sampling with later challenge, recovery, and causal manipulation can test whether developmental history explains response dynamics beyond cumulative exposure or a single persistent abnormality. If simpler persistent-variable or cumulative-exposure accounts explain the observations equally well, the additional complexity of the framework is not warranted.

The contribution of the present framework is to provide an experimentally tractable account of how developmental experience may become incorporated into the organization of a changing biological system and thereby alter the conditions under which later experience is processed. By shifting the question from where an early experience is permanently stored to how developmental history changes the architecture through which future inputs are received, transformed, and regulated, the framework provides mechanistic hypotheses that can be separated, tested, refined, or rejected. This reframing preserves the possibility of persistent developmental influence without requiring persistence of a single physiological state and places the explanatory burden on measurable developmental trajectories, causal mechanisms, and response dynamics.

## Data Availability

The original contributions presented in the study are included in the article/supplementary material, further inquiries can be directed to the corresponding author/s.
